# Epidermal and fibroblast growth factors incorporated polyvinyl alcohol electrospun nanofibers as biological dressing scaffold

**DOI:** 10.1038/s41598-021-85149-x

**Published:** 2021-03-11

**Authors:** Amnah Asiri, Syafiqah Saidin, Mohd Helmi Sani, Rania Hussien Al-Ashwal

**Affiliations:** 1 Department of Biosciences, Faculty of Science, Universiti Teknologi Malaysia, 81310 Johor Bahru, Johor Malaysia; 2 IJN-UTM Cardiovascular Engineering Centre, Institute of Human Centered Engineering, Universiti Teknologi Malaysia, 81310 Johor Bahru, Johor Malaysia; 3 Department of Clinical Sciences, School of Biomedical Engineering and Health Science, Universiti Teknologi Malaysia, 81310 Johor Bahru, Johor Malaysia; 4 Advanced Diagnostics and Progressive Human Care (Diagnostic) Research Group, Health and Wellness Research Alliance, Universiti Teknologi Malaysia, 81310 Johor Bahru, Johor Malaysia

**Keywords:** Biotechnology, Biomaterials, Drug delivery

## Abstract

In this study, single, mix, multilayer Polyvinyl alcohol (PVA) electrospun nanofibers with epidermal growth factor (EGF) and fibroblast growth factor (FGF) were fabricated and characterized as a biological wound dressing scaffolds. The biological activities of the synthesized scaffolds have been verified by in vitro and in vivo studies. The chemical composition finding showed that the identified functional units within the produced nanofibers (O–H and N–H bonds) are attributed to both growth factors (GFs) in the PVA nanofiber membranes. Electrospun nanofibers' morphological features showed long protrusion and smooth morphology without beads and sprayed with an average range of 198–286 nm fiber diameter. The fiber diameters decrement and the improvement in wettability and surface roughness were recorded after GFs incorporated within the PVA Nanofibers, which indicated potential good adoption as biological dressing scaffolds due to the identified mechanical properties (Young’s modulus) in between 18 and 20 MPa. The MTT assay indicated that the growth factor release from the PVA nanofibers has stimulated cell proliferation and promoted cell viability. In the cell attachment study, the GFs incorporated PVA nanofibers stimulated cell proliferation and adhered better than the PVA control sample and presented no cytotoxic effect. The in vivo studies showed that compared to the control and single PVA-GFs nanofiber, the mix and multilayer scaffolds gave a much more wound reduction at day 7 with better wound repair at day 14–21, which indicated to enhancing tissue regeneration, thus, could be a projected as a suitable burn wound dressing scaffold.

## Introduction

Skin regeneration and reconstruction are currently a demand healthcare area due to the global population growth^1^. The skin's external and internal inhibitory factors can delay wound healing and may trigger a chronic wound construction that will increase medical treatment cost. Wound healing is a challenging clinical issue that focuses on wound care management and emphasizes scavenging new treatment approaches^3^. Several conventional wound dressings do not allow the typical physiological environment to facilitate the wound healing process^4^ due to lack of biological activities and responses^5^. Scaffolds are polymeric three dimensional (3D) structures that have the prospect to address the limitation of traditional wound dressing materials. The unique scaffold properties allow cell attachment, cell migration, modify the diffusion of vital cell nutrients and control the behavior of cell phases which exert certain mechanical and biological influences^6^. Various techniques have been utilized to fabricate scaffolds such as solvent casting, freeze-drying, phase separation, gas foaming, electrospinning, fiber bonding, melt moulding, prototyping, powder compaction, supercritical fluid and fiber mesh^7,8^.

Electrospinning is an inexpensive and appropriate way to fabricate nanofiber meshes^9,10^. Numerous factors that affect the electrospinning process, including electrospinning parameters, electrospinning solution and environmental parameters^11,12^. Simultaneously, solution parameters of polymer concentration, solvent, viscosity and solution conductivity, and environmental parameters of temperature and humidity play roles in determining the electrospun fibers' properties. In general, all parameters are identical to be controlled to form smooth and bead-free electrospun fibers^11^.

There are several techniques of electrospinning that have been used by researchers, such as co-electrospinning, adsorption/immobilization, coaxial/multiaxial and emulsion electrospinning^13,14^. These techniques help to control drug release from the high surface area of nanofibers into the applied environment, especially for sensitive molecules or small water-soluble drugs^14^. Co-electrospinning is a versatile and simple technique to obtain nanofibers of different polymers with the ability to load several types of molecules in these structures^15^. Simultaneously, absorption or immobilization is a technique to immobilize molecules on fiber surfaces through covalent linkages that act as a specific molecular cue to control drug release^14,15^. In the coaxial/multiaxial technique, two and more separate solutions are electrospun using core–shell nozzles to maintain the initial bioactivity of molecules^14,16^. However, both absorption/immobilization and coaxial/multiaxial techniques are complicated and require particular attention to material selection, solvent combination and specific volatility^14,16^. The emulsion technique does not require concise apparatus^15^. However, it requests specific properties of molecules, polymers and solvent combination^14^. Therefore, in this study, the co-electrospinning technique was employed to form a polymer -based loaded growth factor (GF) as it is simple and does not require specific chemical properties.

Polyvinyl alcohol (PVA) is a synthetic polymer increasingly utilised in biotechnology, pharmaceutical, food chemistry and medicine, as a film, hydrogel or nanofibers. The significant drivers to using PVA in the medical and pharmaceutical fields are the biocompatibility, processability and hydrophilicity features^17–19^ and the Food and Drug Administration (FDA) approval^17, 20^. PVA is categorized into fully hydrolyzed and partially hydrolyzed^21^. Hence, depending on its type and level of polymerisation,  the PVA's solubility limited its application in the pharmaceutical field, especially for the partially-hydrolysed PVA. Therefore, it has been crosslinked before being used to reduce the hydrophilicity of the PVAin many studies^22–24^. However, the nanofiber the crosslinked PVA nanofiber still exposing the tissue to additional toxicity risks^25,26^. This study used fully hydrogels PVA without crosslinked due to the hydrophobicity that makes it suitable to sustain the extracellular moist nature of the wounds and to degrade slowly compared to the hydrophilic PVA^27,28^. Therefore, PVA was chosen to be used in this study as a polymer matrix to form electrospun nanofibers and to carry two different types of growth factors (GFs).

Tissue development or repair regulated by growth factors proteins along with several cellular processes within the extracellular matrix (ECM)^29^. Among the several families of the cytokines and growth factors^30^ the epidermal growth factor (EGF) considered the first and foremost factor to promote wound healing^31^. It is mitogenic, form various epithelial cells, hepatocytes and fibroblasts^32,32^. It possesses high-affinity receptors that are expressed by both fibroblasts and keratinocytes. EGF utilization is mainly to improve epidermal and mesenchymal regenerations, cell mobility, proliferation and ECM synthesis, which consequently facilitate wound healing^31,33^. At the same time, fibroblast growth factor (FGF)^32^ Acidic and basic are the most known FGF classification^15,32,34,35^. Many functions are attributed to FGF, are due to interaction with cell surface receptors that has intrinsic tyrosine kinase activity, the development of new blood vessels, and the wound repair and hematopoiesis function^34,36^.

As EGF and FGF are playing roles in improving the healing capacity of skin cells and, at the same time, will provide natural remediation, both EGF and FGF in this study were incorporated into PVA to be co-electrospun for the fabrication of wound dressing. The electrospun nanofibers' wettability and elasticity were assessed through a water contact angle (WCA) and tensile test analyses. At a final stage, the nanofibers' biological activity was evaluated through in vitro cell studies with human dermal fibroblast cells and in vivo studies on a mammalian-wound rat model (burn).

## Materials and methods

### Sample preparation

**Figure 1:**
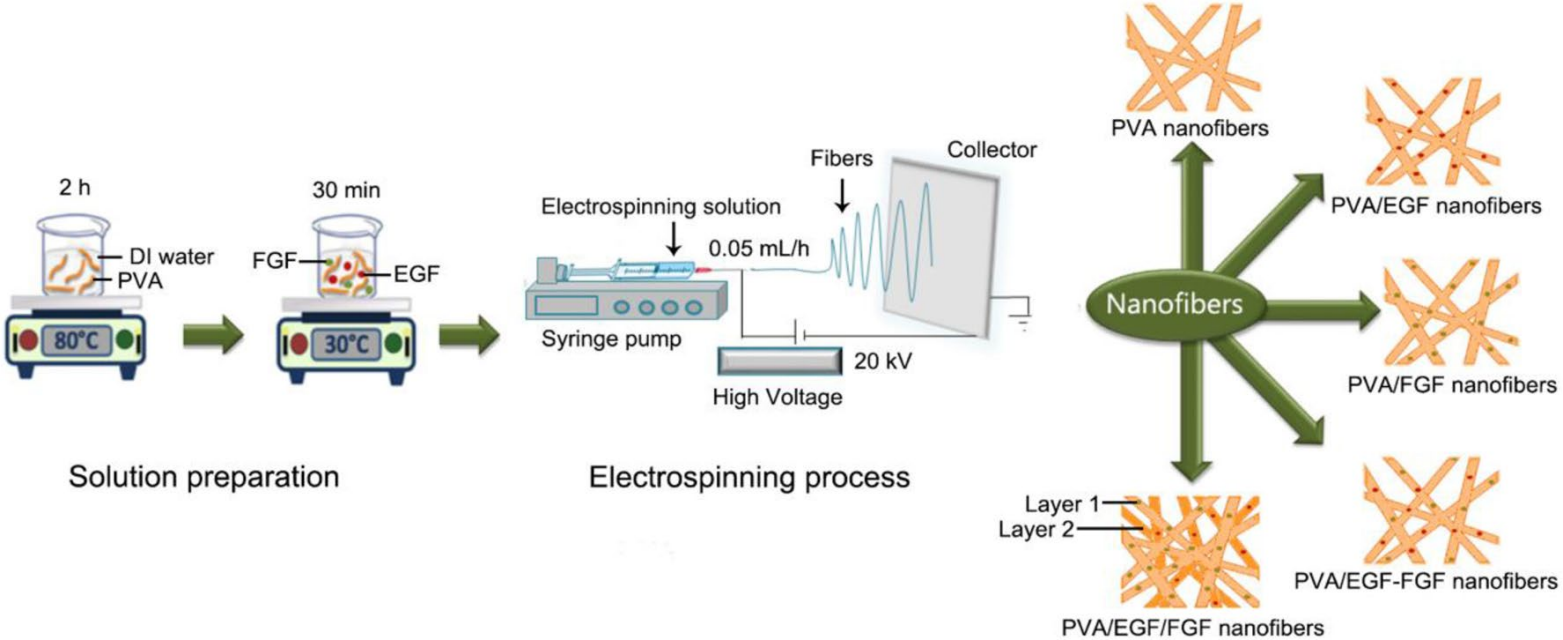
Schematic diagram of electrospun nanofibers process.

This study's materials include a polymer of full-hydrolyzed polyvinyl alcohol (PVA), MW 145,000 (Merck, Japan). Two growth factors of EGF and FGF, phosphate-buffered saline (PBS) and Bovine serum albumin (BSA) (Sigma-Aldrich, Germany, Sigma-Aldrich, USA). The PVA has been dissolved deionized water (DI) at a concentration of 8% to prepare the polymer solution, then stirred at 80 °C for 2 h to obtain a homogeneous polymer matrix solution^37,38^. Amounts of 100 µg EGF and 100 µg FGF were dissolved in BSA/PBS in separate beakers. The growth factor solution mixed with  the polymer solution at a temperature of 30 °C by applying a water bath concept for 30 min^13,31^. For all nanofibers were set at PVA:EGF:FGF, 9.5:0.5:0.5 v/v, respectively. In this study, five membranes were electrospun as stated in Fig. 1, which consisted of pure PVA (control), single layer PVA/EGF, single layer PVA/FGF, mix layer PVA/EGF-FGF and multiple layers PVA/EGF/FGF, which has been fabricated in sequence by creating the layer 1: single layer PVA/FGF, followed by layer 2: single layer PVA/EGF.

This study has utilized an electrospinning setup, as explained in Fig. 1 the used electrospinning machine from NaBond Technologies Co., Limited, Shenzhen, China. The syringe needle was connected to a 35 kV DC supply with a ground collector. For the electrospinning, the prepared solutions were introduced into the syringe and mounted on the syringe pump. The syring to collector distance was set at 15 cm, and a positive voltage of 20 kV was applied at a 0.5 ml/h volumetric flow rate in a room temperature environment^38^.

### Characterization analyses

The nanofibers chemical functionalities were analyzed by ATR-FTIR (L1600301, PerkinElmer, UK) between wavenumbers of 500 and 4000 cm^−1^ at 4 cm^−1^ resolutions. The acquired ATR-FTIR spectra were then annotated and analyzed using Originlab software (Origin Pro 8, OriginLab Corporation, USA)^10^.

Then the nanofibers' morphology was visualized by SEM (JEOL, iT300LV, Japan) at a magnification of 10,000× at an acceleration voltage of 10 kV to view the formation of felt-like nanofibers, possible beads and electrospray fibers. Prior to the SEM observation, the electrospun nanofibers membranes were dried under vacuum (0.1 mbar), and a thin film of gold was coated by using a gold sputter coater (Q150R, Quorum Technologies Ltd, England). The diameter of fibers was measured with mage analysis software (JEOL, iT300LV, Japan). A total of 100 random fibers per image were examined to calculate the mean and standard deviation of fiber diameter^9,10^.

The nanofibers' topography was further identified with AFM (AFM5300E, Hitachi High-Technologies Corporation, Tokyo, Japan) in an intermittent tapping mode of 10 μm × 10 μm area with a back-side aluminium-coated rectangular cantilever. The acquired topography AFM images were then subjected to post-analyses of surface roughness and fiber diameter.

### Wettability analysis

A contact angle instrument have been used to test the hydrophilicity and hydrophobicity (wettability) of the nanofibers(VCA, AST Product Inc, USA). The contact angle measurements were then performed at five different points by dropping 1.5 µL of deionized water on the membranes and measuring the contact angle with a drop shape analyzer.

### Tensile analysis

The elasticity measurement was performed using a texture machine (CT3 Texture analyzer AMETEK Brookfield, United Kingdom). The nanofiber membranes were cut into a dumbbell shape with a nominal 22 mm, 5 mm of length and depth. The membrane thickness was in a range between 0.05 and 0.10 mm. The tensile test was conducted by applying 0.05 N load at 0.2 mm/s test speed according to ASTM standard D5034-95^39^. The measured yield stress (Young's modulus), and the ultimate strength of nanofibers were obtained by stress–strain curve analysis. The results were recorded as an average and standard deviation of three consecutive measurements.

### Cell culture of human dermal fibroblasts (HDFs)

The materials of the cell culture include the human dermal fibroblast cells (HDFs) (ATC-PCS-201-012), 10% fetal bovine serum (FBS) and 1% penicillin/streptomycin, Dulbecco’s Modified Eagle Medium (DMEM). Medium refreshed every 72 h under 95% air, 37 °C and 5% CO2 humid atmosphere. When HDFs reached 80% confluence, cells washed with 0.25% trypsin–EDTA to be collected then re-suspended in phosphate-buffered saline (PBS). The cell passage between 3 and 5 was used. All nanofibers sterilized by ultraviolet (UV) on both sides for 15 min to be used in the in vitro study. Triplicate data for the treated cells were collected, and non-treated cells were used as a control. The cells (HDFs) then seeded and incubated at a concentration of 1 × 105 cells/cm^2^ on each nanofibers membrane, 5% CO_2_ and 37 °C consequently, up to 7 days. The media were changed every 72 h regularly. After 1 h of culture media, the cells attachment on the nanofiber surfaces was observed.

### MTT assay

The produced fibers effect on the cell viability and proliferation was determined by MTT assay 3-(4,5-dimethyl-2-thiazolyl)-2,5-diphenyl-2H-tetrazolium bromide based on color observationafter the incubation at 1, 3 and 7 days. After 1, 3, and 7 days, the media were removed, and 1 ml fresh media were added into each well with 100 μL of MTT solution (0.5 mg/ml) following another incubation for four hours. After that, the media consisting of MTT solution were removed, and DMSO was added to dissolve the formazan products followed by 30 min incubation and with using a microplate reader (Multiskan FC, Thermo Scientific, USA) reading taken at the absorbance of 540 nm. The collected data then processed using the following equation:

1 $${\text{Cell viability }}\left( \% \right) = {\text{Experimental OD}}_{{{54}0}} /{\text{Control OD}}_{{{54}0}} \times {1}00$$

### Cell attachment

The physicohemical properties and roughness of nanofibers significantly influence cell behavior in term of proliferation and adhesion. Using the field-emission scanning electron microscope (FESEM) cell adhesion, the scaffolds' proliferation and morphology images have been analysed^40^. After the HDFs were cultured on the nanofibers and incubated on day 1 and 7, the nanofibers were washed, fixed with PBS (pH 7.4) and 2.5% glutaraldehyde for 2 h consequently. The nanofibers were undergone gradual dehydration in 30%, 50%, 75%, 90% and 100% ethanol for 10 min consecutively. Next, the nanofibers were freeze-dried overnight, coated with gold thin films. The cell morphology and adhesion were visualized under FESEM (SUPRA 35VP, Zeiss, Germany) at 5000× magnifications.

### In vivo wound healing study

The in-vivo study performed according to approved animal care ethical protocols with reference number: UPM/IACUC/AUP-R011/2020 under Institutional Animal Care & Use Committee (IACUC) of Universiti Putra Malaysia (UPM). During the in vivo experiments, we ensured maintaining the animals (rats), i.e. feeding, pain management, locating, and sacrificing rats.  The in-vivo mammalian model was the male Sprague–Dawley (SD) rats, weighting 240–260 g^41,42^, in which a burn created using controlled temperatures and contact times. After staining with hematoxylin and eosin stain (H&E), the obtained histology has been used to characterize changes and  to determine the burn wound healing after exposure to the synthesized PVA-Gfs loaded scaffolds.

### Wounding and preparation of tissue specimens for histological study

Pre-operatively, the rats were kept to adapt to the laboratory conditions for 7 days under a controlled diet and watered adlibitum. Prior to creating the wound, ketamine- HCl and xylazine anaesthesia (100 and 10 mg /kg body weight) were given to the rats under the observation of the veterinary (vet) physician, shaving the dorsal area of the rats. After that, one full-thickness circular burn wound created using a hot circular steel billet (ф = 25 mm; 100 °C, 30 s)^43^. A specialized vet physician has performed the burning procedure to assess the burn thickness and diameter preceding each experimental intervention to reduce the thickness evaluation bias created by the lack of uniform protocol for assessing the extent of tissue injury^44^. After the third-degree burn wound area created, we left the rats wound left to cool down with no medical treatment. Subsequently, the experiment consisted of six groups of rats, and we left the first group without treatment as a negative control (burned). The second group burn was dressed with one layer of the dry sterile scaffold of protein-free PVA as a positive control (burned) while the group three to six were treated with PVA/EGF, PVA/FGF, PVA/EGF-FGF, and PVA/EGF/FGF sequentially and fixed with a surgical suture to prevent the preventing the rats from removing it by scratching or chewing. we used individual cages for rats under a controlled ambient temperature at 23–24 °C with 12/12 h light/dark cycles during the experiments^45^. The control rat killed immediately on day 0; after that, the treated rats were killed on days 7, 14, 21 after the burn and photographs were taken of each wound using a digital camera and measured by a ruler. The data was calculated by adopting the equation mentioned in^46^:

2 $$ \% {\text{ wound closure}} = {\text{ A}}_{0} - {\text{A}}_{{\text{t}}} /{\text{A}}_{0} \times {1}00$$

where A_0_ was the area of initial wound and A_1_ was the area of wound at time.

Three rats of each group were euthanized, and the skin tissue was excised to evaluate skin repair at each time point for histological analysis. The specimens were prepared and washed in saline, fixed in 10% neutral buffered formalin and embedded in paraffin blocks and sequentially sectioned at 5 μm using a microtome. The hematoxylin and eosin (H&E) stain used to estimate the healing phases^47^. Images were taken with a camera on the microscope (T380C-10M, AmScope, USA).

### Statistical analyses

The wound closures measurement has been used to identify the statistical differences among the synthesized PVA loaded GFs. The data were triplicated for statistical analysis purpose. The collected data of cell viability and the wound closure in the in-vivo study analyzed using GraphPad Prism 6.01 software (GraphPad Prism, La Jolla, CA, USA) to calculate one-way ANOVA of variance and Tukey's test for multiple comparisons at P < 0.05.

## Results and discussion

### Chemical composition analysis

**Figure 2:**
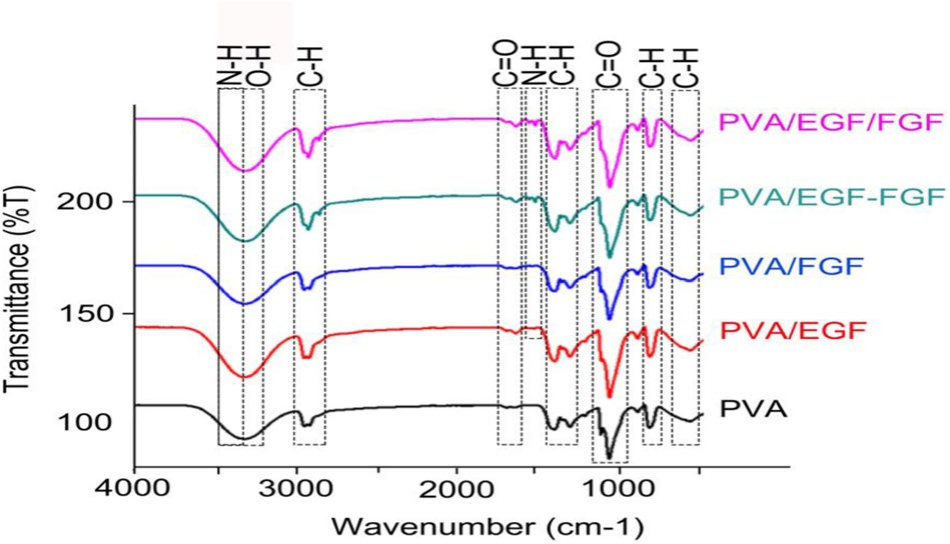
ATR-FTIR spectra of electrospun nanofibers: (a) PVA, (b) PVA/EGF, (c) PVA/FGF, (d) PVA/EGF-FGF and (e) PVA/EGF/FGF.

Figure 2 shows the ATR-FTIR spectra of PVA (control), PVA/EGF, PVA/FGF, PVA/EGF-FGF and PVA/EGF/FGF nanofibers which have been electrospun at similar electrospinning parameters. Functional groups of PVA has been identified in all scaffolds. The presence of hydroxyl (OH^−^) peaks at 3200–3500 cm^−1^ were dominated by O–H stretch and O–H bend, and that existed in the chemical structure of PVA^48^.

The C–H stretch peaks were observed at 2850–3000 cm^−1^, while C–H bend identified at 1450–1470 cm^−1^ and C–H rock peaks were identified at 720–725 cm^−1^. The long hydrocarbon chain in the chemical structure of PVA is dominated to be responsible for the hydrophilic properties of PVA^49^. The presence of C=O stretch and the C-O stretch has indicated the hydrophobic groups of the PVA was then observed in between 1665 and 1710 cm^−1^ and 1000 and 1320 cm^−1 ^, consequently^50^.

The PVA nanofibers incorporated with GF demonstrated the presence of amine groups, N–H stretch at 3300–3500 cm^−1^^38^ overlapping with the OH^−^ groups. Another identical GF peaks of N–H bend are clearly shown only on the GF incorporated membranes at 1580–1650 cm^−1^^51,52^ which clarify the existence of GF in the nanofiber membranes.

### Morphological structure assessment

**Figure 3:**
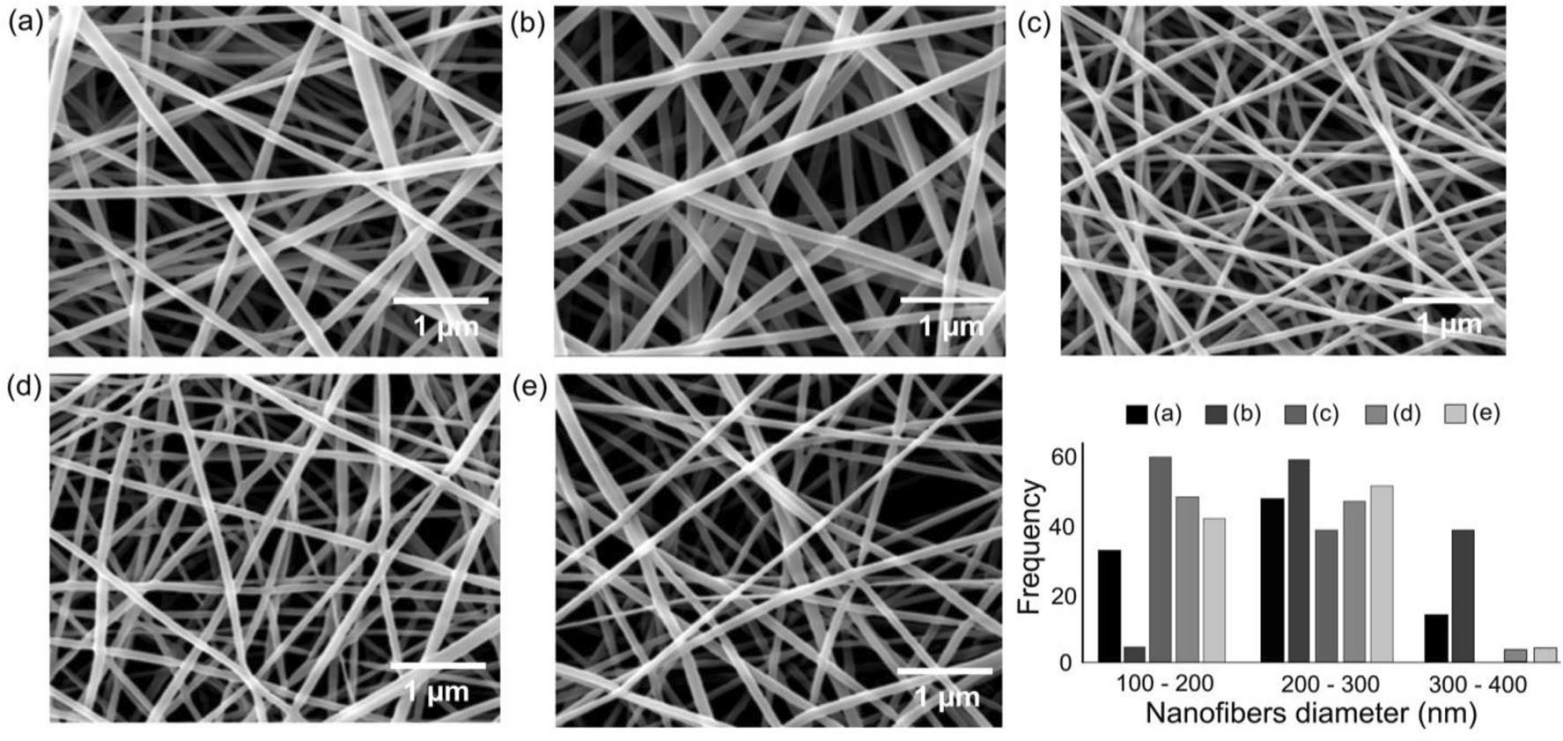
SEM morphologies and fiber diameter histograms for (**a**) PVA, (**b**) PVA/EGF, (**c**) PVA/FGF, (**d**) PVA/EGF-FGF and (**e**) PVA/EGF/FGF.

The SEM visualized the surface morphology, structure and diameter of the electrospun Nanofibers. We found that all nanofibers were constructed in continuous long protruded nanofibers, imitating porous meshes. Figure 3 shows the uniform nanofibers without the bead and electrospray feature for all membranes. The nanofibers' average diameters were counted and illustrated in Fig. 3, where the PVA membranes were constructed of 230 ± 59 nm diameter nanofibers.

The mean diameters for the PVA/EGF and the PVA/FGF electrospun nanofibers were counted to be 286 ± 60 and 198 ± 37 nm, respectively. The molecular weight of GFs was capable of increasing or decreasing fiber diameter through the adjustment in solution viscosity^53,54^. In this study, the low molecular weight of FGF (16–34 kDa) compared to EGF (4.6–74 kDa)^55,56^ has led to the reduction in fiber diameter.

The incorporation of both EGF and FGF produced the nanofiber diameters of 205 ± 50 nm for the PVA/EGF-FGF and 218 ± 52 nm for the PVA/EGF/FGF. These two membranes, mix and multiple GFs, were deposited with the range of nanofiber diameter between of the PVA/EGF and the PVA/FGF. Despite  the molecular weight, other parameters are also affecting the diameter of fibers including voltage, the  distance between needle tip and collector, nozzle diameter, solution conductivity, environmental humidity and environmental temperature^11^. Decreasing the nozzle diameter and increasing the applied voltage^57^, the distance between the tip and the collector^58^, solution conductivity^59^, environment temperature, as well as environment humidity, will reduce the fiber diameter^11^. However, in this study, all electrospinning parameters were kept constant to maintain electrospun nanofibers' ejection.

### Surface roughness analysis

**Figure 4:**
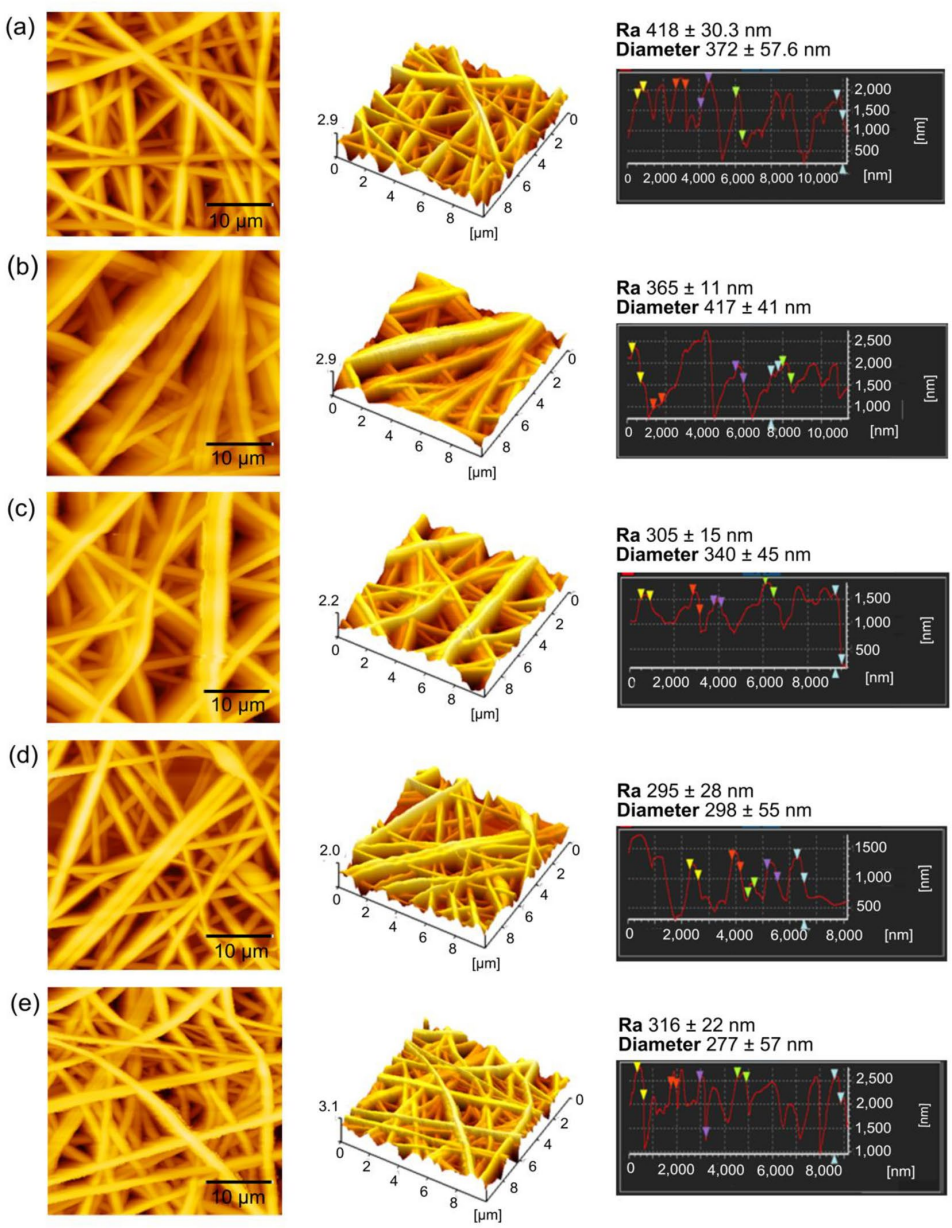
AFM topography images and surface roughness post-analysis of (**a**) PVA, (**b**) PVA/EGF, (**c**) PVA/FGF, (**d**) PVA/EGF-FGF and (**e**) PVA/EGF/FGF.

The surface roughness analysis was performed on the topography AFM data, as shown in Fig. 4, where the PVA nanofibers were recorded with 418 ± 30 nm surface roughness. Decrement of surface roughness was observed on the PVA/EGF and the PVA/FGF with the values of 365 ± 11 nm and 305 ± 15 nm, respectively. The surface roughness was further reduced on the PVA/EGF-FGF with 295 ± 28 nm roughness and the PVA/EGF/FGF with 316 ± 22 nm roughness. There was no significant difference in the surface roughness data between PVA/EGF-FGF and PVA/EGF/FGF membranes, signifying that the incorporation of FGF and EGF, either through the electrospun of mix solution or multiple layers, did not impact the nanofiber roughness. Ahlawat et al*.*^60^ mentioned the capability of low surface roughness to assist cell adhesion and proliferation mechanically. In this study, the low surface roughness of GFs incorporated membranes could provide a medium for cell interaction to induce cell integration and proliferation.

The fiber diameter was further verified through the post-analysis of AFM topography data. The fiber diameter value for all nanofiber membranes (Fig. 4) are identical to the increment and decrement trends of fiber diameter observed through the SEM analysis. However, the values were higher due to the back–and-forth cantilever retraction during the scanning process, cause the data projection to be wider. From the post-analysis results, the incorporation of both FGF and EGF into the PVA nanofibers led to the reduction of fiber diameter with the acquired data of 298 ± 55 nm and 277 ± 57 nm for the PVA/EGF-FGF and PVA/EGF/FGF, respectively.

### Wettability analysis

**Figure 5:**
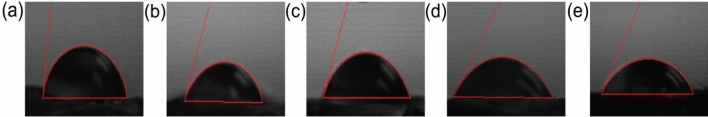
Surface contact angles of different electrospun nanofibers: (**a**) PVA, (**b**) PVA/EGF, (**c**) PVA/FGF (**d**) PVA/EGF/FGF(M), and (**e**) PVA/EGF/FGF(L).

The perspective of wettability properties is preferable for cell attachment, migration and proliferation due to the hydrophilic interaction between cells and material surfaces^61^. The PVA nanofibers produced contact angle data of 83 ± 0.88°, approaching the hydrophobic properties, as the PVA is constructed of C=O and –CH_3_ hydrophobic groups^62,63^. The incorporation of GFs cause the nanofiber membranes to be more hydrophilic with the records of 66° ± 2.98° and 66.08° ± 0.88° for the PVA/EGF and the PVA/FGF, respectively. The decrement of contact angle values was mainly caused by the hydroxyl and amine groups in the GFs, which tailored the properties of the nanofibers towards greater wettability^64^. The combination of EGF and FGF have further reduced the contact angle data to be 53° ± 2.05° for the PVA/EGF-FGF and 57° ± 2.19° for the PVA/EGF/FGF. The combination of both GFs has tuned the wettability of nanofiber surfaces to be higher compared to the singular GF incorporated membranes. These results are identical to the measurements in previous studies^65,66^ which testified high wettability records on the GFs incorporated polymer nanofibers (Fig. 5).

### The tensile analysis

**Figure 6:**
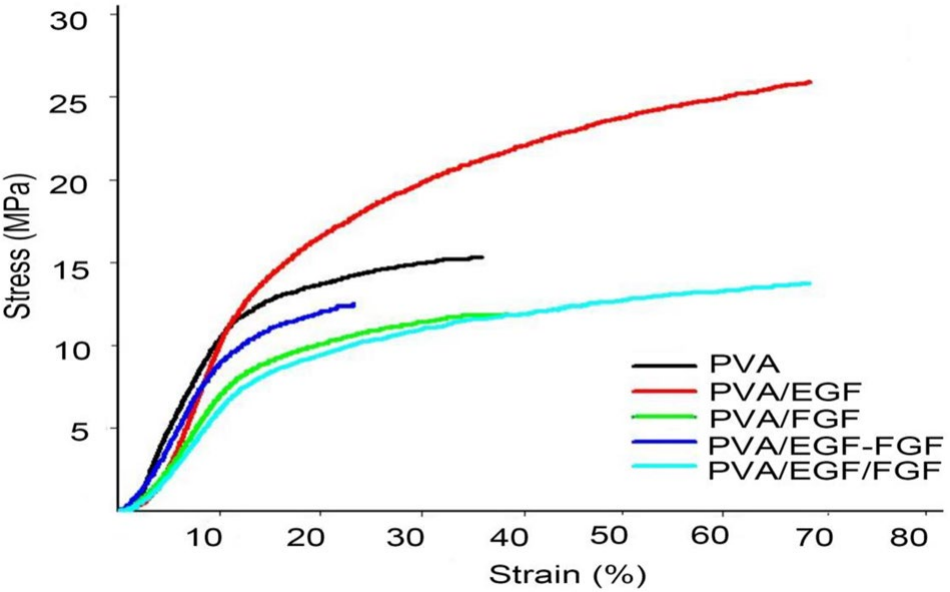
Stress–strain curves of (**a**) PVA, (**b**) PVA/EGF, (**c**) PVA/FGF, (**d**) PVA/EGF-FGF and (**e**) PVA/EGF/FGF.

The stress–strain curves of the PVA, PVA/EGF, PVA/FGF, PVA/EGF-FGF and PVA/EGF/FGF nanofibers under tensile loading data are arranged in Table 1 and plotted in Fig. 6. The Young’s modulus of PVA nanofibers was reduced to 17 MPa, when the FGF was incorporated within the PVA matrix compared to Young’s modulus of pure PVA nanofibers 33.89 MPa.

**Table 1:** Mechanical properties of electrospun PVA, PVA/EGF, PVA/FGF, PVA/EGF-FGF and PVA/EGF/FGF nanofiber membranes.

Electrospun nanofibers	Young’s modulus	Yield strength	Ultimate tensile stress
PVA	34	11.2	15.4
PVA/EGF	36	12.0	26.0
PVA/FGF	17	8.0	12.0
PVA/EGF-FGF	18	8.8	9.2
PVA/EGF/FGF	20	7.2	13.8

In contrast,Young’s modulus of PVA nanofibers was increased to 36 MPa when the EGF was incorporated within the pure polymer membrane. The properties of FGF, which derived from the classification of natural fibers and elastin, have increased the elasticity of the electrospun membrane, which affected the material strain, thus decreasing Young’s modulus value^67^. The combination of FGF and EGF in constructing the PVA/EGF-FGF and the PVA/EGF/FGF cause further reduction of Young’s modulus, between 18 and 20 MPa. The nanofibers (Young’s modulus) in the range of preferable properties of the human skin, 15–150 MPa^68^, which projects a view on its utilization for skin tissue engineering application.

### MTT assay

**Figure 7:**
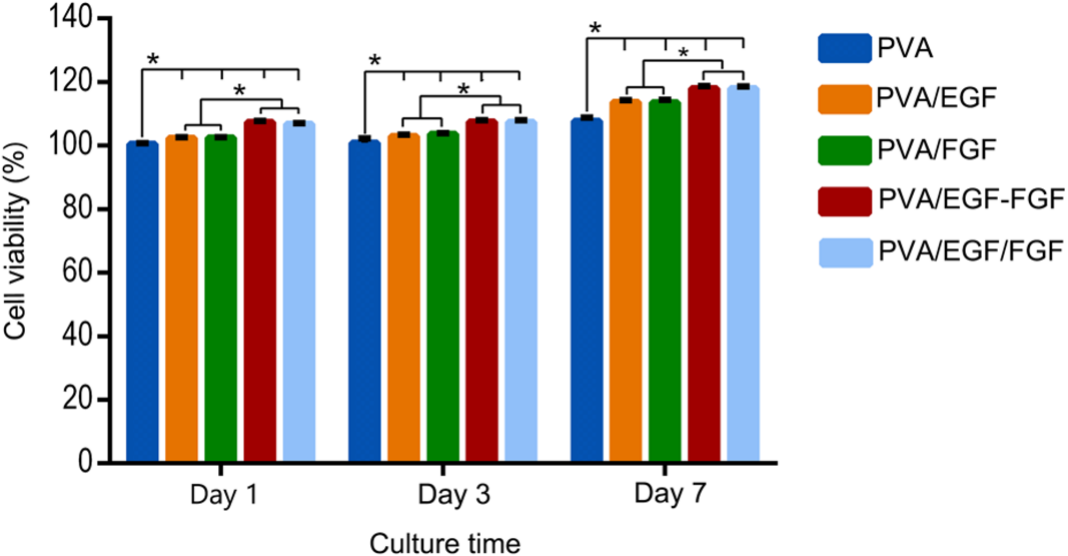
MTT assay data of HDFs after 1, 3 and 7 days cultured on nanofibers.

The in vitro study of HDFs goal s to evaluate the synthesized scaffolds compatibility to be implanted in the biological environment. Cell attachment and proliferation are important factors in designing scaffolds that can enhance and present regeneration of tissue^69^. Figure 7 shows the proliferation of HDFs and cell viability and the nanofibers of PVA, PVA/EGF, PVA/FGF, PVA/EGF-FGF and PVA/EGF/FGF at day 1, 3, and 7.  A similar density of culture was seeded on all nanofibers to evaluate the cellular proliferation by MTT assay. All nanofibers were biologically active with HDFs and enhanced cell proliferation. All nanofibers did not show cytotoxic effect due to the cells viability remains more than 70%,  according to Catanzano et al. and Cerchiara et al. ^70,71^. The cell proliferation trend has increased, starting with slow growth at day 3 to fast growth at day 7 (*P* < 0.05).  On day 1, the observed cells showed a significantly higher proliferation on the GFs incorporated nanofibers than the PVA with significantly greater proliferation on the PVA/EGF-FGF and PVA/EGF/FGF compared to the PVA/EGF and PVA/FGF.  On day 3, a similar trend was observed, while on day 7, the more visible trend was projected with the highest HDFs proliferation on the PVA/EGF-FGF and PVA/EGF/FGF. That indicated the EGF and FGF effect in stimulating the fibroblast cell growth, which was observed much more at 7 days of the analysis with that may indicate the activity of both GFs^64,72^.

### Cell attachment

**Figure 8:**
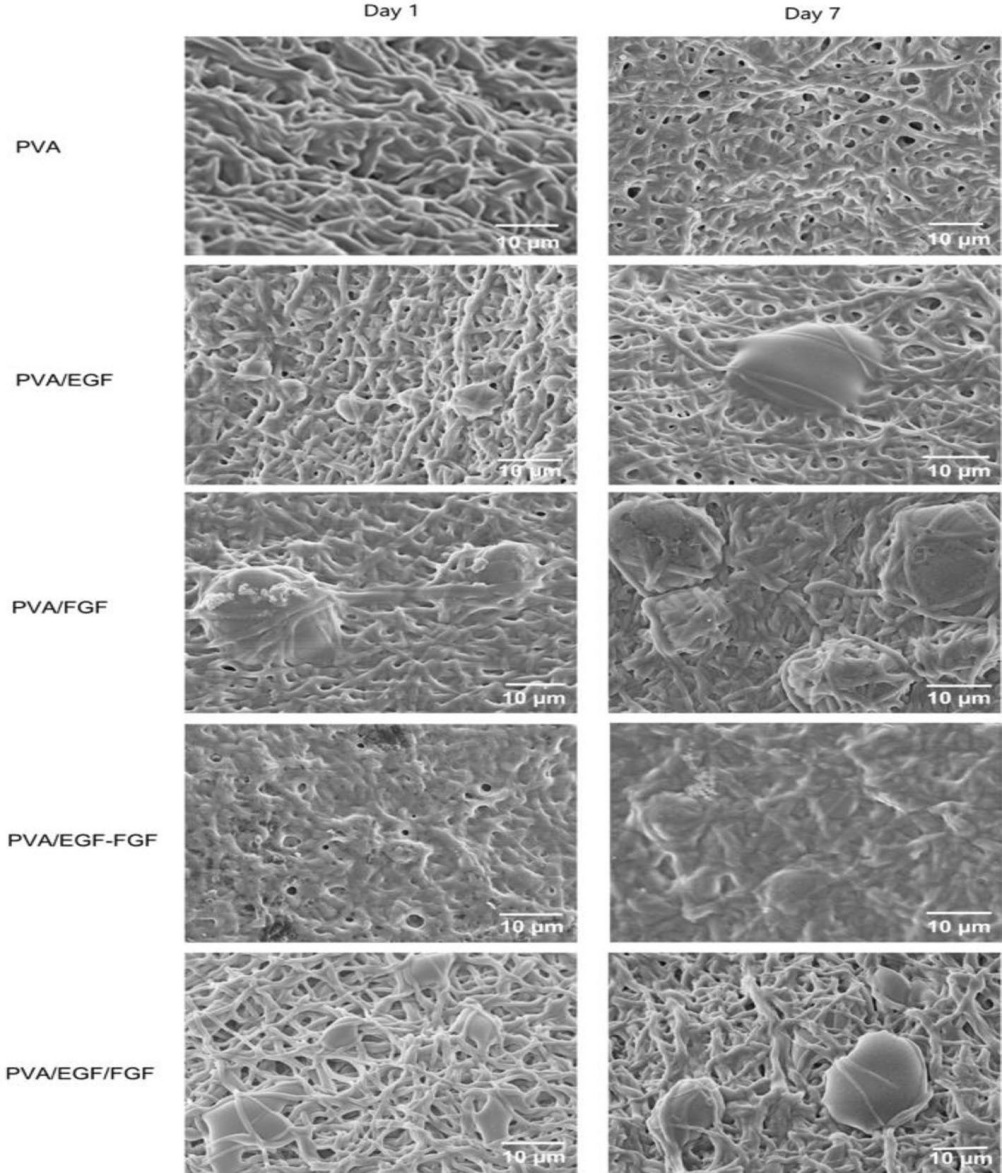
FESEM images of HDFs after day 1 and 7 cultured on nanofibers.

Figure 8 presents the images of the HDFs surface and cells features (morphology), viewed under FESEM to identify the early adhesion of HDFs on the nanofibers on day 1 and 7. The images show that the HDFs were attached and disseminated more on the GFs incorporated nanofibers at respective days with different attachment trend than  the PVA nanofibers. On day 1, the HDFs were slightly adhered and spread in a flat and focal form. The filopodia were elongated and stretched, clutching the porous structure of nanofibers. The HDFs were found in a small cluster on the PVA/EGF, while a  more significant cell cluster was found on the PVA/FGF. On the one hand, the GFs mixture in the PVA/EGF-FGF cause the cells to attach in a small cluster following the trend on the PVA/EGF due to the direct contact between EGF and cells. On the other hand, a big cluster was viewed on the PVA/EGF/FGF due to FGF exposure to the cells, as the EGF layer was at the bottom.  On day 7, the cells were significantly  interacted and spread on the GFs incorporated nanofibers due to continuous EGF and FGF releases, according to Bahadoran et al. and Lin et al.^69,73^. It is clear that HDFs were attached to the PVA nanofibers at day 7 without spreading on the nanofiber surface, which explicated as a passive adhesion due to complex physicohemical interaction between the cell membrane and the polymer surface^69^.

### In vivo wound healing study

**Figure 9:**
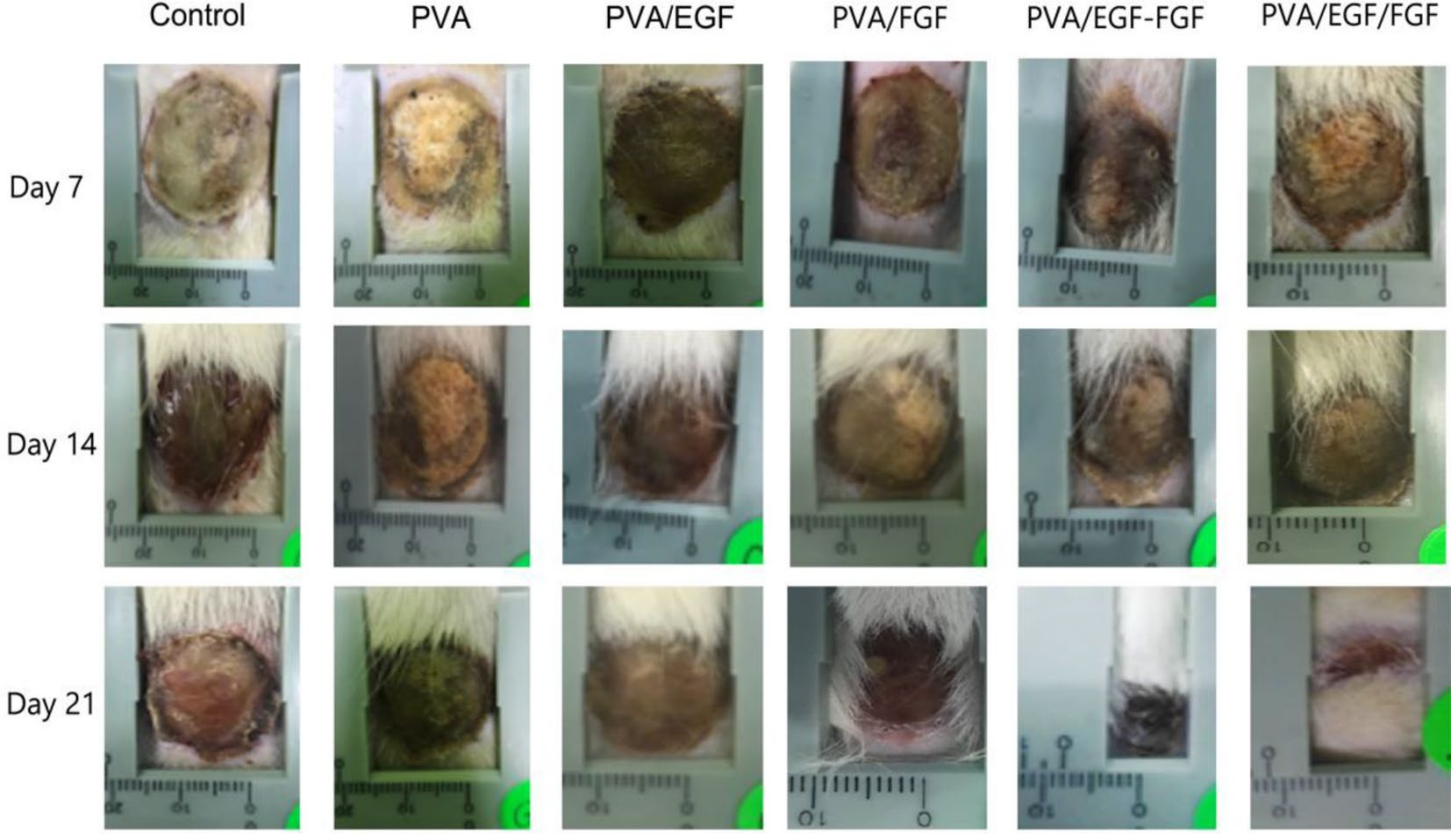
Wound closure rate of fill thickness burn wound treated with various nanofibers.

The clinical evaluation of the wound characteristics and closure is shown in Fig. 8 following the visual assessment of burn depth^74^. Post-treatment observation and the clinical evaluation of the treated rats continue until the 21st day as it is the survival rate average of the burn-wound models due to the application of this study purpose^75^. The process of burn-wound healing of the in-vivo experiment in PVA/EGF, PVA/FGF, PVA/EGF-FGF, and PVA/EGF/FGF nanofibers groups at 7th, 14th, and 21st days have been thoroughly assessed. We have identified the features of the full-thickness burn tissue characterized by the dry, leathery dark brown, and we followed the wound closure within the same period of the healing compared to all the GFs loaded Nanofibers. The burn-wound healing effects of PVA/EGF, PVA/FGF, PVA/EGF-FGF and the PVA/EGF/FGF nanofiber has been visually the confirmed, compared to the positive control (PVA) treated rats and negative control (burned only rats). It was visually found that there were marginal changes in the burn-wound size in all experimental groups at 7, 14, and 21 days’ (Fig. 9). It was found that although there were marginal differences in the burn-wound closure rate of the negative control and positive control, the best closure was shown in the experimental groups at 14 days and 21 days with the mixture and layered PVA/EGF-FGF, and PVA/EGF/FGF sequentially of the healing period, and the best burn-wound closure rate was shown in PVA/EGF/FGF (Fig. 9). These results are most probably related to growth factors’ activity^76,77^.

**Figure 10:**
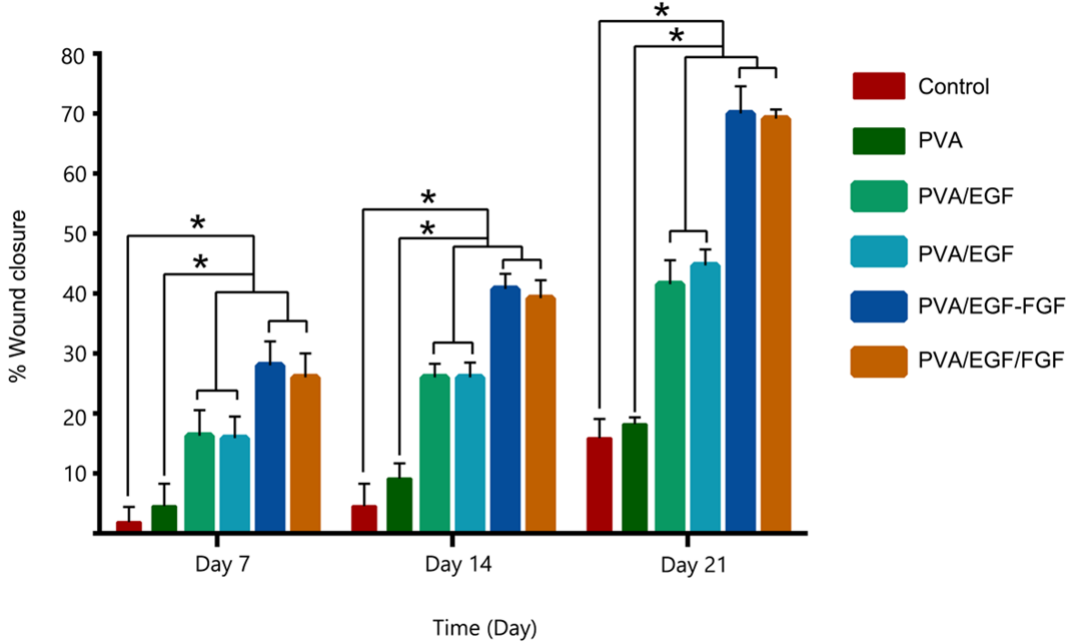
The percentage of wound closure treated with different nanofibers.

The percentage of the wound healing (closure) measurements for the experiment groups are presented in Fig. 10. All the PVA/EGF, PVA/FGF, PVA/EGF-FGF, and PVA/EGF/FGF nanofibers could enhance burn wound healing compared to control and PVA groups, while the control group show sign of hemorrhage and inflammation in the wounds. Consistently, the quantitative data represented in Fig. 9, showing the same trend of growth with some variations in the results between the mixtures and layers PVA/EGF-FGFand PVA/EGF/FGF. PVA/EGF-FGF and PVA/EGF/FGF nanofibers displayed high rapid recovery of the wounds contrasted to that of only EGF or FGF nanofibers, which imputed the combined effects of EGF and FGF. Figure 9 detected that on the 21st post-burn wound day, the closure rate of the PVA/EGF-FGF and PVA/EGF/FGF groups (70%, 69.2%) were significantly higher compared with the PVA/EGF (41.6%), PVA/FGF (44.7%), PVA (18%), and control (15.9%). Moreover, it was found that PVA-GFs loaded nanofiber was a good scaffold for burn-wound healing as compared to PVA only nanofiber. The observed healing in the PVA only fibers might be due to the characteristics of the nanofiber that assist in working as an extracellular mesh^76,77^. This finding indicates the EGF and FGF inside scaffold that promoted the epithelialization, collagen synthesis, and tissue regeneration. However, the variations found to reflect the capability of the utilized PVA in this study to carry the proteins to the burned tissue within an acceptable period can not confirm the design influence as the difference exists but can not differentiate the most influencing design. Despite this observed inconsistency, we can elude that the type PVA utilized in this study has no limiting effect on its capability to be used as a protein carrier, as shown in Figs. 9 and 10.

### Histological analysis of burn-wounds

**Figure 11:**
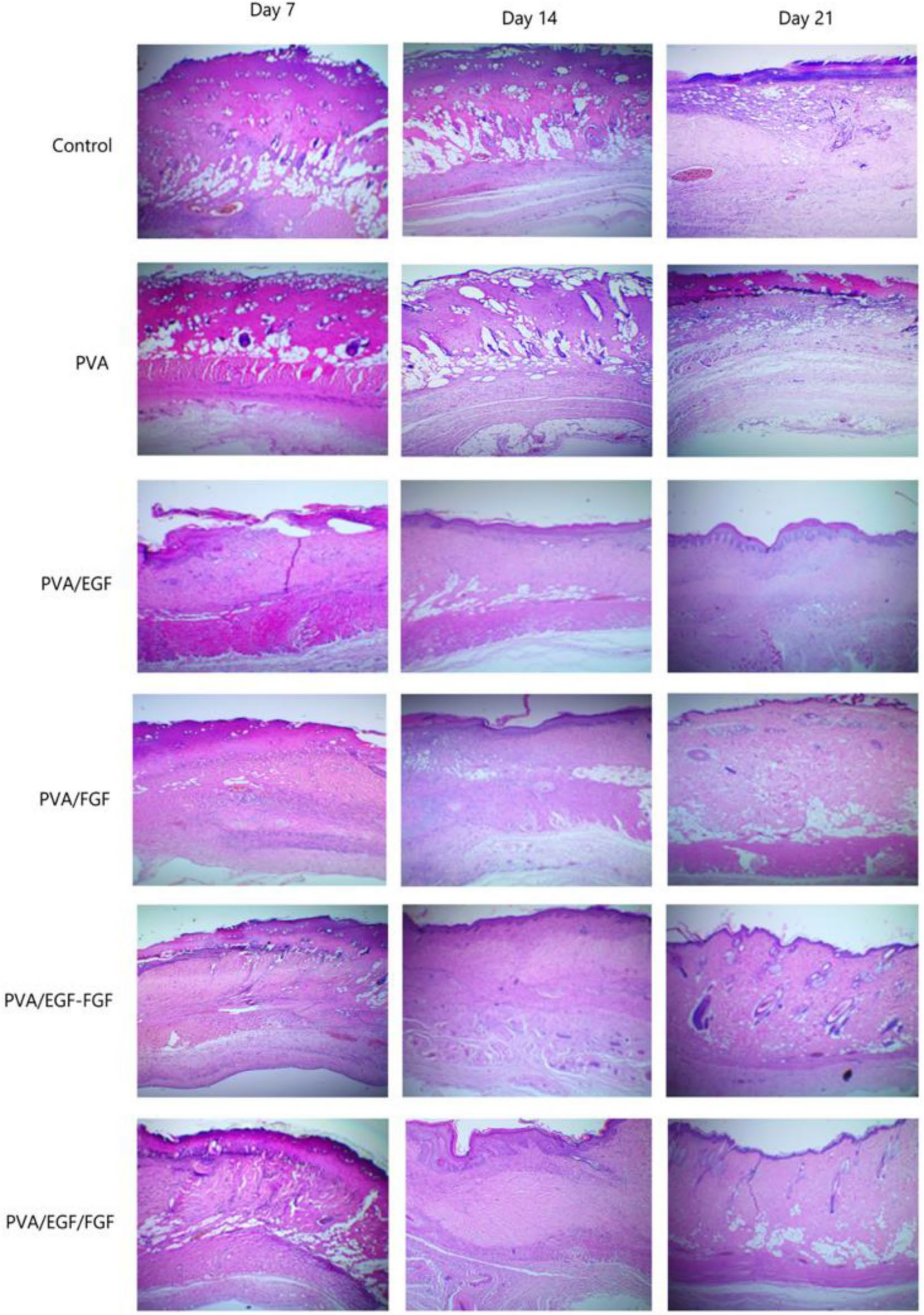
Histological sections of full thickness burn wound treated with various nanofibers (×40 magnification).

Histological analyses of the burn healing process were performed by H&E stain on day 7, day 14, and day 21in Fig. 11. On day 7, the epithelial tongue was grown, which show the new regenerated epidermis in PVA/EGF, PVA/FGF, PVA/EGF-FGF and PVA/EGF/FGF whilst the necrosis was seen in control and PVA group. At Day 14, stratified epidermal layers grew more in PVA/EGF, PVA/FGF, PVA/EGF-FGF and PVA/EGF/FGF. The low level of necrosis was shown in the control and PVA group. On day 21, the papilla basement membrane was obviously seen in PVA/EGF while PVA/FGF few sebaceous glands were observed. The neovascular structure was seen in PVA/EGF, PVA/FGF, PVA/EGF-FGF and PVA/EGF/FGF. PVA/EGF-FGF and PVA/EGF/FGF were observed sebaceous gland and hair follicles and show healthier skin on day 21. However, PVA and control groups show a newly regenerated epidermis with necrosis in the control group. The control of infection in the healing wound is very important for a good progressive wound closure, specifically in the burn-wound healing process^78^. From the finding in this study, it is likely that the wound closure not interrupted with infections and has lead to a proliferation in the dermal cells and accelerated the healing effect. The finding was investigated that PVA/EGF-FGF and PVA/EGF/FGF can enhance collagen synthesis, re-epithelialization and vascularization with a short time of healing due to EGF stimulate epithelialization in human wound repair, and FGF promoted the proliferation of dermal fibroblasts^79^.

## Conclusion

In this study, we fabricated and characterized Growth factors incorporated PVA electrospun mats with single, mix and multiple layers as burn-wound healing scaffold. The incorporation of EGF and FGF in PVA nanofibers, either through single, mix or multiple layers, led to the detection of O–H and N–H from the chemical groups of GFs. All nanofibers were electrospun without the bead and spray feature, where the combination of EGF and FGF has reduced the fiber diameter. The wettability, surface roughness and mechanical properties of the GFs incorporated PVA nanofiber membranes were also improved, which fit its utilization as a biological dressing scaffold.

The synthesized fibers have been examined for feasibility to be used as a wound-healing dressing. The burn-wound healing effect was examined by digital camera observation and histological analyses. The GFs incorporated PVA nanofibers display no cytotoxic effect and cell proliferation within the nanofibers' structure regarding in vitro studies. The in vivo study evaluatin shows the GFs incorporated PVA nanofibers accelerated the burn wound healing process that enhances epithelialization and proliferation of dermal fibroblasts.
